# Solid Waste Generation Rate, Composition Analysis, and Proposed Management Plan: A Case Study of Main Market Centers of Bahir Dar City

**DOI:** 10.1155/tswj/8504268

**Published:** 2025-03-16

**Authors:** Amare Kassawe, Eshetu Getahun

**Affiliations:** ^1^Faculty of Chemical and Food Engineering, Bahir Dar Technology Institute, Bahir Dar University, Bahir Dar, Ethiopia; ^2^Bahir Dar Energy Center, Bahir Dar Technology Institute, Bahir Dar University, Bahir Dar, Ethiopia

**Keywords:** Bahir Dar City, market center, solid waste composition, solid waste generation rate, solid waste management plan

## Abstract

Effective municipal solid waste management is a critical aspect of urban development. This study investigated the waste generation rate, composition, and current solid waste management plan in Bahir Dar City, Ethiopia. A two-stage random sampling technique was implemented. Data collection involves onsite data collection, structured questionnaires, and semistructured interviews. The result indicated that retail trade emerges as the leading sector, generating nearly half of the total solid waste (49%), and the food service sector stands second. In terms of solid waste type, food waste constitutes a significant portion of the waste stream, with a daily generation of 10,817.51 kg. The paper and cardboard waste in the market centers were the second waste, accounting for 762.684 kg per day. The amount of plastic waste generated per day ranges from 157.946 to 493.253 kg, and because of its high volatile matter content (68.95%), it might be used to produce energy. Food waste and yard waste had a high moisture content of 63.25% and 40.14%, respectively, which makes them ideal for composting and biogas production. Among the seven study sites, Kebele 04 had a huge waste generation, which was 47% of the total waste generation, indicating a spatial disparity in waste production in the city. The results highlighted the immense potential for waste reuse and recycling, emphasizing the circular economic opportunities associated with sustainable waste management practices. The findings contribute valuable insights to urban planners and policymakers to implement sustainable solid waste management plans in the country.

## 1. Introduction

Globally, the population in urban areas is increasing at an alarming rate, hence an enormous volume of waste is generated every day [1–4]. One of the main problems facing developing countries, especially in cities that are expanding quickly, is the volume of municipal solid wastes [5–7]. The main causes of this are uncontrolled urbanization and population expansion. The urban environment is severely deteriorating as a result of uncontrolled urban growth and high population rise [8]. Thus, solid waste management (SWM) regulates the generation, handling, collection, transportation, and storage of solid wastes as well as their processing and disposal in a way that safeguards public health, economics, engineering, conservation, and public opinion's best practices [9].

Reports indicated that solid waste may be a useful resource if used properly, but if it is not handled well, it can have major negative impacts on the environment and public health [1, 10]. In many low- and middle-income countries, SWM takes up a significant portion of the total recurrent municipal budget in cities. Local governments frequently struggle to provide everyone in need with appropriate and dependable services despite the heavy budgetary burden. According to the World Bank and USAID, municipalities in developing countries often allocate 20%–50% of their available municipal budget to SWM [11].

Various SWM strategies are available today. The greatest way to handle a community's solid waste crisis is through integrated solid waste management (ISWM), which uses a range of tactics and services to regulate the urban waste stream [12]. With a focus on optimizing resource use efficiency, ISWM is a strategic approach to the sustainable management of solid wastes from all sources and in all respects. It includes generation, segregation, transfer, sorting, treatment, recovery, and disposal in an integrated manner [13–15]. An efficient ISWM system takes into account the best practices for solid waste management, recycling, and prevention to safeguard the environment and public health. In ISWM, the most suitable waste management practices are chosen and combined based on an assessment of the local needs and conditions. Waste prevention, recycling, composting, burning, and disposal in properly planned, built, and managed landfills are the main ISWM activities. All of these activities, which are covered in this and the other fact sheets, require meticulous planning, funding, collection, and transportation. With an emphasis on sustainability and minimizing the negative effects on the environment, ISWM is a comprehensive method that covers all phases of waste treatment, from creation to disposal. Source reduction, recycling, composting, and waste-to-energy programs are all part of this comprehensive approach. To maximize waste management systems, ISWM combines technological, social, economic, and environmental factors. It highlights how crucial it is to involve the community, recover resources, and reduce the environmental impact of disposing of solid waste [16].

In developing countries like Ethiopia, an estimated 30%–50% of solid waste produced in urban areas is left uncollected [10]. The primary steps in the SWM process are the generation and storage of solid waste, which is followed by collection, transportation, and transfer of solid waste. Next comes cleaning the streets, recovering recyclables, treating and disposing of solid waste, and lastly gathering input to keep an eye on and assess the SWM procedures [15]. The primary obstacle to waste management in Ethiopia is the small number of waste segments that can be recycled on an informal basis. For personal benefit, only a small percentage of people separate exchangeable and saleable wastes; most rubbish is not separated. In certain regions, the population is even less than half that. The gathering happens erratically, and it might not even happen once a week at worst. The frequency of the service could be once a week, twice a week, or even less frequently [17]. Ethiopia has numerous regions, cities, and towns, all of which have huge populations and produce a lot of waste in the urban.

Bahir Dar City is one of the highly populated regional cities in Ethiopia that generates a substantial amount of solid waste. Bahir Dar is one of Ethiopia's fastest-growing and most expansive cities. Because Bahir Dar is situated near Lake Tana and the Abay River, which are home to a variety of flora and animals, it is also well-recognized that the city is naturally attractive. Furthermore, one of the most popular tourist destinations in the country is Lake Tana, which is home to numerous monasteries [7]. Bahir Dar City has six subcities and 26 kebeles with seven known market centers: one in the Belay Zeleke subcity, two in the Dagmawi Menelik subcity, one in the Fasilo subcity, one in the Gish Abay subcity, one in Atse Tewodros subcity, and one from Tana subcity. According to the report, Bahir Dar City generates 192 tons of solid waste daily, and 27% of it comes from commercial areas. The commercial sector is still expanding rapidly, and the business sector is growing quickly; three market centers have opened in the past 5 years alone.

With a 6.6% annual population growth rate, Bahir Dar is one of Ethiopia's fastest-growing and most expansive cities [1]. The rate at which solid waste is generated rises in tandem with population growth. The city of Bahir Dar produces 192 tons of solid waste per day, with commercial districts contributing 27% of this waste, according to the Bahir Dar City Structural Plan (BDSP) study [18]. As a result, the environment is more polluted (air, soil, groundwater, and surface water). People are susceptible to a variety of diseases due to environmental pollution. Thus, to lower the impacts associated with it, efficient management of the created waste is crucial [19].

Inadequate governance and policy frameworks in Bahir Dar City make the city's SWM problems even worse. However, there have been several major institutional and organizational changes recently in the Bahir Dar SWM system. The collection and transportation of municipal solid waste was delegated to a private waste management company by the municipal authority of Bahir Dar in 2008 [11]. In the past few years, a large number of micro and small businesses have been established to provide waste precollection services. These businesses are paid by municipalities or the individual beneficiaries to gather and transport waste to municipal waste disposal sites, which helps to close the gaps in the collection and transportation of waste, but still, it is not sufficient [1].

Planning a municipal SWM system successfully requires having accurate information about the rates and type of waste generated. Unfortunately, one of the main causes of Bahir Dar City's inadequate SWM is the absence of timely data on waste composition and generation. Although studies on waste management in homes and apartments are common, to obtain a complete picture of the situation, it is essential to collect data on the rates and types of waste generated in the commercial, institutional, industrial, street sweeping, and hospital sectors. Despite being largely ignored, the commercial sector contributes significantly to waste generation. Since there are no suitable landfills or alternative disposal options in Bahir Dar City, it is imperative to determine the kind and volume of waste produced by the business sector. There is currently insufficient waste collection and disposal capacity in Bahir Dar due to the city's rapid expansion and growing waste creation. Furthermore, comprehension of the amount and makeup of waste becomes essential for the creation of suitable strategies and plans for the future as Bahir Dar City develops into a significant business hub and tourist destination center. Without properly understanding the amount and type of trash produced, it is impossible to develop ambitious strategies for waste reduction, reuse, and recycling. This is especially true given that marketplaces are major suppliers of solid waste, which can be both a burden and a useful resource.

Bahir Dar City struggles to manage its solid waste without a well-integrated system. The optimization of waste reduction, recycling, and sustainable disposal is hampered by a lack of a comprehensive methodology. This field of study looks at possible ways to establish a complete waste management framework and determines how the lack of an integrated approach exacerbates SWM issues in the city [3]. In Bahir Dar City, there is urgent concern over the growing amount and shifting mix of solid waste. Increased solid waste output with a more diverse composition has been brought about by rapid urbanization and population rise. As waste management systems are adapted to accommodate the changing nature of solid waste in the city's expanding urban landscape, research into the mechanisms causing this surge is necessary [19].

The various types of solid waste sources include residential, commercial, institutional, building and demolition, public facilities, industrial, and agricultural treatment plant sites [20]. The identification of types of solid waste and composition in the city's marketplace is becoming a crucial factor in the creation of waste management plans. Determining the origins, compositions, and patterns of solid waste generation is, therefore, essential to creating efficient waste management plans [21]. The physical and chemical characterization of solid waste properties such as moisture content, elemental composition, specific energy (calorific value), and density is very important to designing an effective waste management plan [22].

In Bahir Dar City, the unorganized waste collectors dominate recycling. Since they acknowledge the positive impact of their behavior on waste reduction, the local authorities tacitly tolerate it even if there is no legal basis for it. Approximately 10–15 jobless waste collectors work at the landfill, recovering recyclable and reusable materials such as glass, metal, plastic, and textiles, which they then sell to brokers or a private waste company (mostly recyclable plastics) [11]. However, integrated recycling of solid waste is minimal in cities. The majority of them are dumped on open fields. However, the local government has created a green vision organization that creates compost from organic waste and offers training to any willing and interested person or group. The recycling potential of solid waste is a key aspect of sustainable waste management [3]. It examines how technology has improved recycling procedures and assesses whether recycling programs are financially feasible. It is essential to comprehend the potential for resource recovery through recycling to reduce trash sent to landfills, preserve natural resources, and lessen the environmental effects of the extraction and manufacture of raw materials [23]. To the best of the authors' knowledge, the main market center of Bahir Dar City has insufficient data regarding waste source type, waste generation rate, composition, and recycling method to support the development of efficient waste management plans in the city. This study insights the mechanism of MSW segregation at the source, a comprehensive solid waste collection and recycling mechanism that covers all waste generators. The study result is also used as a benchmark for policy makers and regulators to implement standard SWM plan and strategy to control the solid waste in the city.

Therefore, the overall objective of this study is to quantitatively and qualitatively analyze the current SWM practices, quantify solid waste generation rate and composition, and propose a waste management plan for the solid wastes generated in the main market centers of Bahir Dar City.

## 2. Materials and Methods

### 2.1. Description of the Study Area

Bahir Dar City is the capital of the Amhara National Regional State in the Federal Democratic Republic of Ethiopia. It is located at 11⁣^″^38⁣′ N 37⁣^″^10⁣′ E on the southern side of Lake Tana (where the Blue Nile River starts). The altitude of the city is about 1801 m above mean sea level. According to the BDSP population study report, Bahir Dar had a total population of 313,997 in 2017 and 455,901 in 2022 and is expected to continue to grow fast currently [24].

**Figure 1:**
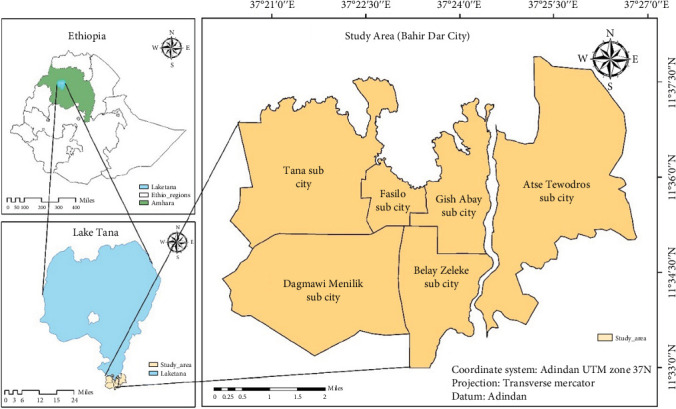
Location of the study area and subcities of Bahir Dar City (authors' own figure).

**Figure 2:**
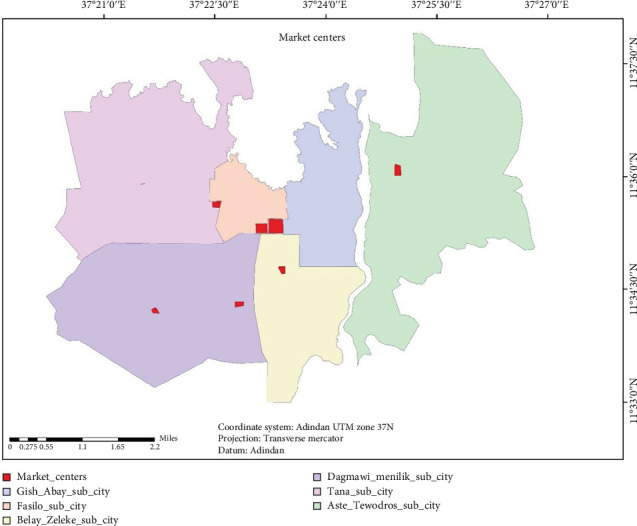
Map that shows the market centers on each subcity in Bahir Dar City (authors' own figure).

Bahir Dar City comprises six subcities, as illustrated in Figure 1, and seven major market centers, as indicated in Figure 2. These include one market center from Tana subcity, commonly named “Kidanemihret gebeya”; two in Dagmawi Menelik subcity, commonly named “Gaja mesk gebeya” and “Abunehara gebeya”; one in Fasilo subcity, commonly named “Kebele 4 gebeya”; one in Gish Abay subcity, commonly named “Kebele 12 gebeya”; and one in Atse Tewodros subcity, commonly named “Abaymado gebeya.”

### 2.2. Materials

#### 2.2.1. Waste Sampling Equipment

Selecting appropriate equipment for waste sampling can indeed be challenging due to the uncertainty surrounding the physical characteristics and nature of the wastes being sampled. The variability in waste composition makes it difficult to determine the most suitable equipment for separation, homogenization, and containerization. Factors such as viscosity and particle size can further complicate the sampling process. It is important to consider that the physical characteristics of waste can change under different environmental conditions, including temperature, humidity, or pressure. These variations can affect the behavior and properties of the waste, making it crucial to select equipment that can accommodate such changes. To ensure accurate sampling and prevent contamination or alteration of the waste, it is recommended to use nonreactive materials for the sampling equipment. The chosen materials should neither introduce any additional substances into the waste nor modify its chemical or physical properties. This is essential to maintain the integrity of the waste sample and obtain reliable data.

In line with these considerations, plastic bags (such as kurtu pestal) were selected as the sampling equipment. Plastic bags made of appropriate materials can effectively contain waste without interfering with its composition or characteristics. Plastic bags are generally resistant to chemical reactions and can provide a suitable barrier between the waste and the sampling container, preventing cross-contamination.

### 2.3. Data Collection Procedure

The collection of detailed and organized data is crucial when collecting waste samples [25]. To ensure accuracy and proper identification, detailed labeled tags were attached to the sampling containers. Additionally, all relevant information such as sampling locations, containers, tanks, and additional equipment, as well as markings/labels, were fully documented in logbooks. Photographs were also taken to record this information, serving as a useful reference throughout the waste sampling processes. During the data collection phase, a meticulous data collection sheet was meticulously prepared to ensure the systematic gathering of information. The process involved collecting solid waste samples from two market centers, specifically during the closing time of the sampled shops. A total of 36 samples were collected over seven consecutive days, commencing on Monday and concluding on Sunday. To facilitate waste collection from various sources, plastic bags of the same sizes were provided, each labeled with a unique code. These bags were distributed to each source, allowing them to store their waste during their daily working hours.

Following the collection of each sample, the sample was weighed to quantify the amount of solid waste. Subsequently, a detailed sorting process was implemented to classify the waste according to predefined categories. This rigorous approach to data collection is aimed at capturing the composition of solid waste in the selected market centers over the specified period. To streamline the subsequent analysis, the collected data was digitized daily into Excel. This digitization process not only facilitates efficient storage and organization of the data but also ensures that it is readily accessible for in-depth analysis. Overall, the comprehensive nature of the data collection, spanning different days and considering various market centers, provides a robust foundation for gaining insights into the patterns and characteristics of solid waste in the specified locations.

In addition to the data collected from waste samples, structured questionnaires and semistructured interviews were conducted with the commercial center community and various stakeholders. These methods provided an opportunity to gather information, opinions, and insights from the stakeholders, enabling a comprehensive understanding of waste management issues. Furthermore, focused group discussions were organized to encourage interactive and in-depth conversations on specific topics related to waste management. By employing these data collection techniques, including detailed documentation, waste sampling, weighing, record-keeping, and engaging stakeholders through questionnaires, interviews, and focused group discussions, a comprehensive and informative dataset was gathered to support waste management analysis and decision-making processes.

Regarding the structured questionnaires, the researcher opted for a digital method. This decision was motivated by several factors. Firstly, using digital questionnaires is a time-efficient approach as it eliminates the need for manual data entry and analysis. Responses can be automatically recorded and stored, reducing the overall workload and potential errors associated with manual processes. Additionally, digital questionnaires can save costs compared to traditional paper-based methods. There is no need for printing, distributing, and collecting physical questionnaires, which can be resource-intensive. Digital surveys also allow for easier dissemination to a larger audience, potentially increasing the response rate and the diversity of respondents. Although most of the questionnaire was digital, some preprinted questionnaires were also employed in some areas.

### 2.4. Sampling Techniques and Sample Size

Due to the heterogeneity in the population under research, a two-stage random sampling technique was chosen as the sampling technique for this study. A heterogeneous unit was subdivided into strata or nonoverlapping groups, and using this sampling technique, each stratum was specified so that its interior is fairly homogenous. Out of the seven market centers, four of them (“Silassie gebeya,”,“Gaja-mesk gebeya,” “Abunehara gebeya,” and “Kidanemihret gebeya”) seem homogenous in their size form and nature of trade exist in the area. They are located out of the city center around residential neighborhoods with medium to small in size. Hence, they can be on the same strata. The rest of the market centers (“Kebele 4 market center,” “Kebele 12 market center,” and “Abaymado gebeya”) are relatively medium and large with a more diversified nature, which makes them line on the same strata. Thus, in this study, there were two strata to make more homogenous the heterogeneous diversified population size.

To assess accurately the population under investigation, various approaches were employed for larger market centers such as Kebele 04, Kebele 12, and Abay-Mado. For these centers, data was obtained from multiple sources, including the trade and industry office, commercial mall records, and other relevant stakeholders. This ensured a comprehensive understanding of the population size and composition in these bustling market centers. However, the situation was different for smaller markets, as many of them did not possess trade licenses, making it challenging to obtain reliable data from government offices. In light of this challenge, the researcher employed an alternative and effective approach. Recognizing that almost all traders in these markets pay a security bill, the researcher leveraged this payment record as a proxy for estimating the population size. By collaborating with the security committee responsible for collecting these payments, the researcher was able to extract the necessary numbers from the bill payment list. This approach provided a valuable source of data that allowed for a reasonable estimation of the population size in these smaller market centers, despite the absence of official government records.

The study divided the market areas into two strata, and these two strata have their subdivision.  Table 1 summarizes the population size, strata division, and the selected sampling market centers.

The sample size was determined by considering resource limitations and representativeness of the sample. Note that market centers solid wastes are wastes that originate in wholesale, retail, commercial buildings, stores, warehouses, and other nonmanufacturing activities [26]. Kebele 04 market center was selected from strata one purposively due to its most diversified nature, and it can represent the others. Silassie market center was diversified when compared to the other small market centers. Both the selected markets were nearby and this made the data collection easier.

Based on the degree of accuracy required in the findings, the minimum number of samples to be collected should be decided. The variation in results across samples serves as another factor in determining the number of samples. In situations when the variability may not be well defined, estimations of the variability may be employed, or this information on the variability may come from literature reviews, pilot studies, past waste composition studies, or waste composition studies finished in other jurisdictions [27].

The number of samples required to achieve a precision objective can be calculated using the following equation [27]:

 $$\begin{array}{c} n=\frac{z^{2}*\delta^{2}}{E^{2}} \end{array}$$

where *n* is the number of samples. *z* is the *z*-statistic for the desired confidence level and the number of samples. *δ* is the standard deviation of the population (from previous studies with comparison). *E* is the precision requirement or margin of error (i.e., one-half the range of the confidence interval).

The required level of precision was defined and then examined in terms of available budget and resources. The selected confidence level and allowable marginal error (*E*) were 95% and 5%, respectively. To apply the above formula, the value of the standard deviation of the population was needed. Therefore, since there was no available data on standard deviation, the estimation comes from the literature. The *z*-statistic can be calculated from the confidence level and for a 95% confidence level *z*-statistic value is 1.96. The margin of error can be predetermined to 4%.

$$\begin{array}{cc} \; & n1={\left(1.96*\frac{0.056}{0.04}\right)}^{2}=8\\ n2={\left(1.96*\frac{0.143}{0.04}\right)}^{2}=49 \end{array}$$

Therefore, the minimum number of sample sizes estimated should be the average of the two which is 28 samples. The value of each stratum was determined based on proportional allocation. In proportional allocation, the sampling effort in each stratum is directly proportional to the size of the stratum [28]. About 36 samples were taken since the minimum sample size was 28. Sample size allocation for a 36-sample size is summarized in Table 2.

Based on proportional allocation, a total of 32 samples were collected from Kebele 04, ensuring a comprehensive representation of the area. Additionally, to further enhance the representativeness of the data, an additional four samples were collected specifically from the bustling Sillasie market center. This approach is aimed at capturing the unique characteristics and dynamics of this specific commercial hub.

The second stage of stratification is a crucial step in the research methodology, as it involves subdividing the first stage strata into different commercial activities. Taking into consideration the valuable input from stakeholders, who possess extensive knowledge of the local trade landscape, the most frequent trade sectors have been identified. These trade sectors encompass a wide range of economic activities, including wholesale trade, retail trade, food services, and various other sectors (offices, game houses, and health facilities). The inclusion of these additional sectors reflects the multidimensional nature of the commercial environment in market centers.

To determine the appropriate sample size allocation for each trade sector, a thorough review of literature, data from governmental offices, and some data that was available, as well as extensive consultation with stakeholders, were conducted. This process allowed for the extraction of percentage proportions that accurately reflect the importance and prevalence of each trade sector within the community as shown in Tables 3 and 4.

### 2.5. Data Analysis

Data analysis is, in short, a method of putting facts and figures to solve the research problem. It is vital to finding the answers to the research question. Another significant part of the data analysis is the interpretation of the data, which is taken from the analysis of the data and makes inferences and draws conclusions. Often, it becomes difficult to deduce the raw data, in which case the data must be analyzed, and the result of the analysis.

Using Excel and the Statistical Package for the Social Sciences (SPSS) software Version 27, the descriptive data analysis on solid waste was conducted, which includes calculating summary statistics, creating visual representations, and organizing information for comprehensive insights into waste characteristics and trends. Photography was used to get visual representation. Additionally, Adobe Illustrator was used for the graphic presentation of labeled trashcan.

## 3. Results and Discussions

### 3.1. Solid Waste Generation Rate

Understanding the quantity and composition of solid waste generated in different areas is crucial for effective waste management and sustainable development. As described in the methodology of the study quantification of solid waste focused on two prominent sampling sites: Kebele 04 and Silassie market centers.  Table 5 presents a comparative analysis of the solid waste quantities recorded at each site. In the “Kebele 04” market center, the major solid waste generators were retail trade shops, accounting for 84.4% of the market. It generated an average of 3070.2 kg of solid waste per day while food services was the second and generated 2159.9 kg of solid waste daily. Wholesale and other trade types rank as the third and fourth solid waste generators with 818.12 kg and 155.41 kg of waste, respectively.

Food waste was the dominant waste type in Kebele 04 market centers (weighing around 4886.2 kg/day), followed by paper and cardboard, recyclable plastics, and textiles (Table 5). As observed in Kebele 04 market centers, the retail trade shops were the primary solid waste generators, followed by food services. Food waste was the most significant waste type, emphasizing the need for effective waste management strategies, particularly in the retail and food service sectors. A similar report indicated that food waste is the dominant waste followed by paper waste in Bahir Dar City [29].

On the other hand, the “Silassie” market center primarily consisted of retail trade establishments, covering 96% of the trade types. Food services accounted for 4%. Food waste was a significant component, amounting to 490.96 kg of solid waste per day. Recyclable and nonrecyclable plastics contributed 3.6284 kg and 3.2161 kg, respectively (Table 5). Other waste types were negligible. It was worth noting that 99% of the waste generated in this market center was recyclable or reusable, with only 1% being disposable waste. The findings suggested that in the Silassie Market Center, the major waste stream was food waste, indicating the need for efficient management and potentially exploring methods for food waste reduction or recycling. The high percentage of recyclable or reusable waste highlighted the market center's potential for implementing effective waste management practices, such as recycling programs, to minimize the environmental impact of the solid waste generated.

#### 3.1.1. Solid Waste Generation Rate in Market Centers

Based on the data obtained from the selected sampling sites, the daily solid waste generation rates were calculated for “Kebele 04” and “Silassie” market centers. These waste generation rates represent the average amount of waste generated per day in these two market centers. Using the solid waste generation rates from the sampled market centers, the waste generation rates for the remaining five market centers were estimated Table 6). This estimation was conducted to approximate the daily waste generation for the entire set of market centers based on the representative sampling sites. Table 6 presents a comparative analysis of the solid waste quantities in each market center including the magnitude and composition of solid waste generated per day.

In Bahir Dar City's market centers, the solid waste composition consists of various components, each requiring specific waste management approaches. Food waste constitutes a significant portion of the waste stream, with a daily generation of 10,817.51 kg, similar to other Ethiopian cities [30]. Implementing initiatives such as food waste reduction campaigns, composting programs, and food donation networks can help divert this waste from landfills and reduce its environmental impact. Yard waste, including grass clippings, leaves, and branches, accounts for 34.218 kg/day. Encouraging practices like composting and mulching can provide a sustainable solution for managing yard waste and promoting soil health. Next to food waste, paper and cardboard waste in the market center amounts to 762.684 kg/day. Plastic waste was a significant issue since every day, 157.946 kg of nonrecyclable plastics and 493.253 kg of recyclable plastics were produced. Textile waste, totaling 447.69 kg/day, necessitates the implementation of textile recycling programs, donation initiatives, and support for sustainable fashion practices. Rubber and leather waste is relatively small at 4 kg/day.

Electronic waste, amounting to 27.43 kg/day, requires specialized management due to its hazardous nature. Establishing e-waste collection points, collaborating with certified recyclers, and raising awareness about responsible e-waste disposal are crucial steps to prevent environmental and health risks associated with improper handling [31]. Glass waste comprises 52.67 kg/day, while metals contribute 60.073 kg. Other waste materials (hair, nail, chemicals, and so on) weigh 185.6 kg/day. Encouraging recycling programs, expanding collection systems, and utilizing recycled materials in manufacturing processes can help conserve resources and reduce waste generation.

#### 3.1.2. Solid Waste Generation Rate Between Market Centers and Different Trade Sectors

The distribution of waste generation across market centers reveals a concentration of the issue in specific areas. Kebele 04 stands out with almost half of the total waste generation, indicating a spatial disparity in waste production. This spatial variation is influenced by factors such as population density, economic activities, or the nature of businesses in each market center.

**Figure 3:**
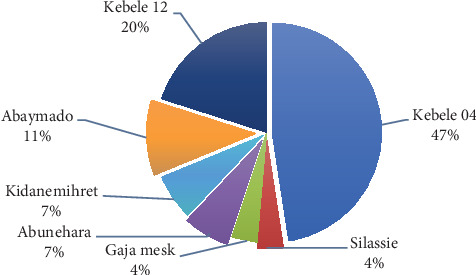
Solid waste generation rate per day of market centers.

The smaller market centers, while individually contributing less to the total waste stream, collectively represent a significant share. This suggests a distributed pattern of waste generation that should be considered in the development of waste management strategies. Understanding the unique waste profiles of each market center is crucial for implementing targeted and efficient waste management practices.  Figure 3 shows solid waste generation between market centers quantitatively.

**Figure 4:**
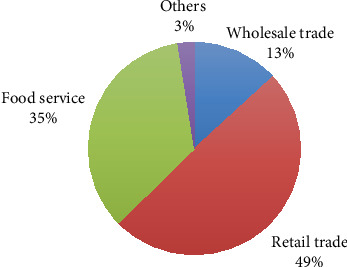
Solid waste generation rate in different trade sectors.

Solid waste generation rates in different trade sectors are presented in Figure 4. Retail trade emerges as the leading sector, generating nearly half of the total solid waste. This dominance suggested that consumer-oriented activities significantly influence the overall waste production in the city. The food service sector follows closely, underlining the importance of businesses involved in food preparation and service as substantial waste generators. Reports also indicated that retailers are the dominant waste generator [12].

#### 3.1.3. Solid Waste Composition Analysis

Numerous variables, including geographic location, population density, consumer behavior, waste management techniques, and cultural norms, might affect the market center's solid waste composition. For example, because small market centers primarily focus on agricultural products, the majority of their waste is food waste, and their waste composition is not very diverse. Reports also indicated food waste composition is also dominant [30]. Large markets, however, have a more varied waste composition. Plastic waste (both recyclable and nonrecyclable plastics), food waste, yard waste, paper and cardboard, textiles, rubber and leather, electronic waste (e-waste), glass, metals, and other materials make up the solid waste of market centers of Bahir Dar City. The waste composition of the Kebele 04 market center and Silassie market center are summarized in Tables 7 and 8, respectively. Too much variation existed in the composition of solid waste among trade sectors. Food waste from agricultural products and food services was enormous, whereas waste from other sources was negligible (Tables 7 and 8). In some industries, such as textiles and related products, cosmetics, household appliances, pepper, and spices, wastes such as plastics, paper, and cardboard were typical, while other wastes are either nonexistent or minimal (Tables 7 and 8). In contrast, hair, nail, and electronic waste were among the waste categories found in beauty salons and electronics stores, which were not typically seen in other trade sectors (Tables 7 and 8).

### 3.2. Current SWM Practice

Market centers are a significant focus for business activity in Bahir Dar City. Nonetheless, the SWM approaches in these market centers significantly differ from one another. Of the seven market centers, only two “Kebele 04” and “Kidanemihret” received regular, independent solid waste collection. The other five, on the other hand, were dependent on weekly waste collection services that were shared with neighbors and street sweepers.

A unique problem exists for market locations without regular solid waste collection. The solid wastes were dumped on the sides of the streets if they were close to arterial or subarterial avenues. The waste was collected and disposed of on the sidewalks for the street sweepers to clear. The marketplaces that were off the main roadways, however, had to wait for the waste collectors to show up. The accumulation of waste and the creation of offensive odors were caused by this collection delay and street sweepers cleaned the street. The problem of waste accumulation exists even in market centers that do receive regular solid waste collection. The market centers were forced to dispose of their waste in neighboring areas, especially close to the streets, due to a shortage of suitable waste-collecting containers. The waste keeps piling up until the municipal solid waste collectors show up to take it out. Since no entity has been explicitly defined as being in charge of this duty, it is unclear who is responsible for keeping the local streets in these marketplaces clean. As a result, it is challenging to handle the waste in many of these marketplaces since they lack adequate waste collection containers. The state of SWM in Bahir Dar City's marketplaces is worrisome overall. Market centers that regularly collect solid waste as well as those that are not have difficulties keeping their environments clean. The problem is made worse by the lack of an obvious entity in charge of street cleaning in the community. Furthermore, market centers are forced to rely on stopgap measures like placing waste close to the streets until municipal solid waste collectors due to the lack of appropriate waste collecting containers, which impedes effective waste management operations, can pick it up.

Nonetheless, there is potential for substantial value to be produced from the waste in these market areas if it is properly gathered and sorted. The Dream Light PLC waste collecting company has already begun making plans for producing charcoal briquettes from waste. On the other hand, they have acknowledged that it can be difficult to get enough food waste for composting. This problem results from improper separation and sorting of the solid waste at the sources.

An important problem with waste treatment, especially in the wake of closure, was discovered by the digital survey that was distributed using Google Forms. When administering the questionnaire, there was a notable agreement among respondents about the efficiency of the various collection tools. Despite the difficulties in managing waste, the majority of respondents indicated that, given the right resources, they would be willing and conscious to manage waste properly. This implies that the lack of proper waste collection services and inadequate equipment, rather than people's willingness to manage waste appropriately, is the real problem.

These results highlighted how critical it is to solve the lack of equipment and upgrade the infrastructure for waste management, especially in smaller marketplaces. A cleaner and more sustainable environment may be promoted by giving people and the local community access to enough resources and skills to improve waste-handling techniques. Furthermore, the knowledge collected from the questionnaire answers might direct focused activities to deal with particular issues and enhance waste management procedures in the locations under study.

### 3.3. Recycling Potential of the Solid Waste

It is necessary to evaluate the recycling technologies already in use in the country, keeping in mind the particular materials previously mentioned, to ascertain the recycling potential in Bahir Dar City's marketplaces. The buildings, machinery, and resources devoted to recycling operations have a major impact on how effective recycling is. It is significant to remember that the potential for recycling can change over time as new technologies appear, infrastructure gets better, and public perceptions of recycling change. For this reason, regular evaluations and updates of Bahir Dar City's recycling capability are necessary to guarantee efficient and long-lasting waste management procedures in the marketplaces [32]. Currently, thick plastic packaging, some plastic cans, various plastics, paper and cardboard, metals, glass, food trash, and yard waste are all recyclable in Ethiopia. These items are categorized as recyclable or reusable according to Ethiopia's current recycling technology. However, the country does not yet have recyclable textile waste [33].

**Figure 5:**
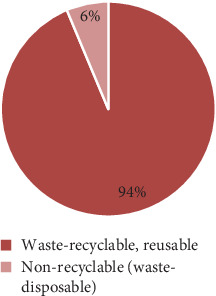
Ratios between recyclable and nonrecyclable solid wastes.

The results of the study indicated that there were two types of waste generated every day: waste that can be recycled and waste that needs to be disposed of as shown in Figure 5. Approximately 12,219.4 kg of waste was generated daily, or 93.69% of the total waste generated was recyclable. Materials including food scraps, yard debris, glass, metals, paper, cardboard, and recyclable plastic were all included in this category. Given the current state of technology, these materials may be recycled. Conversely, waste intended for disposal accounts for 6.31% of all waste produced or around 822.667 kg each day. Waste materials that have limited or no potential for recycling were included in this category. Nonrecyclable plastics, textiles, electrical trash, rubber, leather, and other wastes like hair and nails were a few examples of these materials. These materials need specific methods that might not be easily accessible, or they do not have many possibilities for recycling.

Furthermore, recycling a sizable amount of the generated recyclable wastes, including textiles and hair, would probably be possible provided the country has well-established recycling infrastructure and facilities. Although they are currently being reused if they are stored separately, textiles, such as garments and fabrics, can be recycled using a variety of methods. Mechanical recycling is a popular technique that involves shredding and processing textiles to produce fresh yarns or fibers that can be used to make new textile goods. In addition, methods for chemically recycling textiles are being developed so that their constituent chemicals can be utilized to make new materials or goods. Additionally, human hair may be recyclable [34]. It is reusable and can be gathered in various ways. For instance, salon hair clippings can be used to make hair booms, which are absorbent structures used to contain and remove oil spills. Because hair is so rich in nutrients, it can also be used as a natural fertilizer or to produce compost. To examine the possibility of recycling solid waste in market centers, the average proximate analysis was very important and carried out and the results are presented in Table 9.

Waste made of plastic has a high volatile matter content (68.95%), making it a good candidate for energy production. The volatile components of plastic are easily evaporated or converted into gas by thermal treatment or combustion methods. Unrecyclable plastics can potentially be used to generate electricity. The proper infrastructure and facilities are necessary for the production of electricity from plastic waste. Regretfully, there may not be the infrastructure required to efficiently turn plastic trash into electricity except in the capital city of Ethiopia, Addis Ababa. However, food waste has a high moisture content (63.25%), and yard waste has a moisture level (40.14%), which makes it ideal for composting and biogas production. The natural process of composting entails the controlled breakdown of organic substances, including food waste. Food scraps and yard trash contain moisture, which gives microorganisms the ideal conditions to break down organic material into nutrient-rich compost and biogas. This suggests that the necessary infrastructure and knowledge are in place to manage food waste and turn it into compost and biogas. To properly utilize food waste and yard trash for composting and biogas, the issue of unsorted waste needs to be addressed. The availability of high-quality food waste for composting and biogas can be increased by putting into practice appropriate waste segregation procedures, educating the public, and promoting responsible disposal of yard waste and food waste. This will encourage the creation of nutrient-rich compost for landscaping or agricultural uses and further improve the sustainability of waste management techniques in the research area.

In conclusion, the outcome showed that there was a significant chance for solid waste to be recycled and reused. This potential stems from the ability to find materials in the waste stream that can be processed, recycled, or rescued. Reusable materials can be diverted from landfills, such as glass, textiles, and some types of plastic, helping to promote a more environmentally friendly method of waste management. In addition, the study highlights a large market need for waste that may be recycled and reused. Economic opportunities and incentives are generated by this need for recycling-focused waste management techniques. Industries and companies looking to recycle materials, resulting in the creation of a circular economy where waste is valued as a resource, provide a possible market for the recovered resources. Furthermore, the handling and collection stage is a crucial bottleneck in the waste management process, according to this study. Inadequate infrastructure, careless handling, and ineffective collecting methods all lead to environmental pollution, health issues for the public, and underuse of important resources found in the waste stream.

Generally, the results indicated that solid waste that can be recycled and reused is often in high demand. However, there was a disconnect, though, as the waste produced in market centers was not getting to the businesses that needed it. By bridging this gap and enabling a more effective waste movement from market centers to businesses, the proposed SWM strategies hope to improve total waste utilization and sustainability in Bahir Dar City. Opportunities for recycling plastic waste include waste-to-energy methods, chemical recycling, mechanical recycling, and bio-based polymers as a substitute to address Ethiopia's solid waste issue. Among the waste conversion techniques, waste to energy is the suitable method and the waste is directly converted to energy using simple pyrolysers, which can be easily affordable for the communities.

Effective SWM has major advantages for the economy and the environment in addition to being essential for preserving a clean and healthy environment. Effective SWM systems provide significant financial benefits like job creation, cost savings, and the growth of a circular economy in addition to helping to create a more sustainable future. A skilled workforce is needed for waste management tasks like collection, sorting, recycling, and disposal, which create jobs in a variety of industries. More job possibilities may arise as a result of the growth of new companies and industries brought about by the extension of recycling initiatives and the construction of recycling infrastructure. Furthermore, the recycling industry supports domestic industries and commerce by selling and exporting recovered materials, which boosts local economies. Businesses, households, and governments may all save a lot of money by using effective waste management techniques. Communities can cut expenses related to waste collection, transportation, and landfill disposal by minimizing waste generation. Since recycling and composting are frequently less expensive than conventional waste disposal techniques, they can help lower waste management expenses. Waste management organizations can also make money by selling recycled products, which gives them another source of income. Taxes and levies related to waste management operations are additional sources of income for governments. Through SWM system optimization, societies can lessen financial burdens, reroute funds to other social and economic objectives, and deploy resources more effectively. Ineffective management of the generated solid waste may lead to problems for society, the environment, and human health [35].

### 3.4. Proposed Management Plan for Solid Waste

The SWM plan strategically focuses on the crucial stages of collection, handling, and sorting to address the main issues with SWM inside market centers in Bahir Dar City. To solve the widespread problem of solid waste collection, this study proposed a waste management plan of sector-specific collection systems, encourages source reduction, establishes frequent and regular collection schedules, and places a strong emphasis on raising awareness. To maximize the enormous potential for recycling and reuse provide extensive education programs in marketplaces to provide awareness of waste handlers, customers, and vendors about the enormous possibilities for recycling and reuse. Stress the financial and environmental advantages of avoiding landfills by using appropriate waste management techniques. Provide specific reuse locations inside or close to the market centers so that locals and vendors can buy and donate reusable goods. Encourage the idea of a circular economy by promoting the resale of goods to extend their useful life. Provide incentive schemes, such as discounts or special recognition, to companies and individuals who actively participate in reuse techniques. Encourage collaborations with nearby companies to build a network of environmentally friendly operations.

Create a market or partner with already-existing recycling businesses to create a marketplace where sellers can directly sell recyclable materials to meet the growing demand for recyclable and reusable solid wastes. Provide a clear and effective transaction structure to promote a steady supply of recyclables. Establish waste exchange programs that allow companies in the market centers to trade recyclables with one another. Promote the development of a network of cooperative relationships for the recycling and upcycling of materials. Businesses that actively participate in recycling activities should be given support and incentives in the form of opportunities for promotion, certificates, and recognition. Investigate joint ventures with recycling businesses to provide recyclable collection services.

Provide specific training programs for waste handlers to improve their abilities in efficiently managing and sorting solid wastes, to mitigate issues that arise throughout the handling and collecting phases. Stress how crucial it is to identify and separate items at the source to reduce contamination. At collection locations, make appropriate infrastructure investments for waste sorting, such as labeled containers and instruments to help waste handlers sort waste effectively. Work with IT companies to investigate intelligent trash cans that enable automated sorting. Use real-time tracking devices, like sensor-equipped bins or GPS tracking, to monitor how well waste is handled and collected. Give waste handlers who regularly follow the right sorting procedures prompt feedback and rewards.

Through education, incentives, and effective infrastructure, this suggested SWM strategy seeks to address market demand for recyclable and reusable goods, maximize the enormous potential for reuse and recycling, and overcome obstacles throughout the processing and collection phase. Modifications may be implemented following the unique attributes and requirements of the market centers. The approach uses several mechanisms in the handling and sorting phase, including specialized sorting stations, bins with clear labels, standardized container types, convenient locations, and intensive awareness campaigns. The management plan is strategically divided into two sectors, responding to the distinct demands of larger and smaller market centers, in recognition of the various nature of enterprises in the market centers.

One noteworthy element that makes waste disposal easier for the community is the color-coded system for containers. Gray/black containers are used for waste that is neither organic nor recyclable, while green containers are used for yard waste, food waste, and other organic materials. Blue containers are used for conventional recyclables like bottles, cans, and plastics, as well as organic waste like paper and cardboard. By streamlining waste management procedures, this logical strategy hopes to promote sustainability in both bigger and smaller market centers. Furthermore, strong waste management policy, regulation law, and enforcement strategy could be implemented in the city to effectively manage the generated solid wastes.

### 3.5. SWM Plan for Larger Market Centers

**Figure 6:**
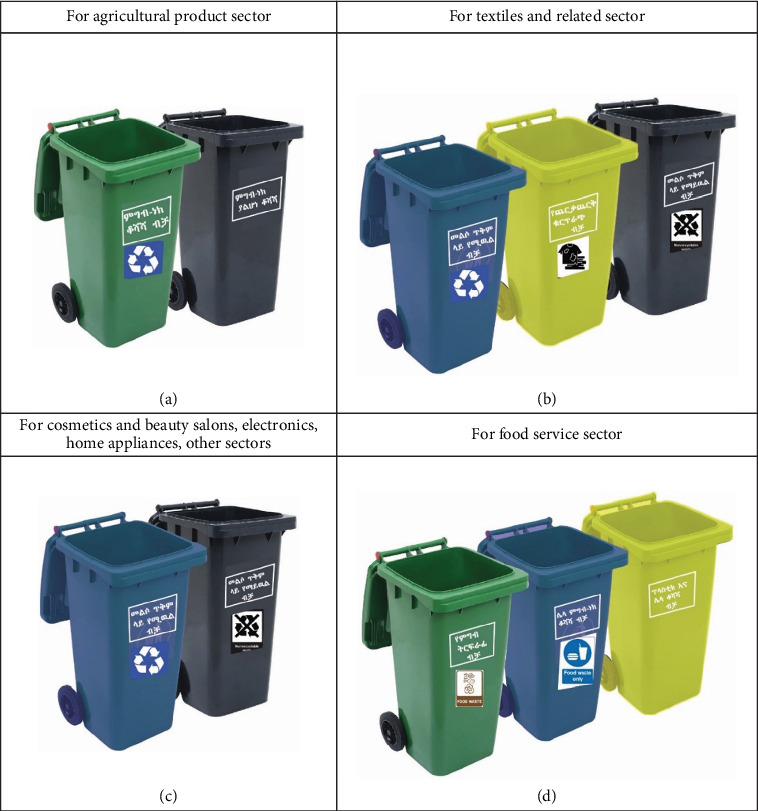
Labeled with local language (Amharic) trashcans for the collection of solid wastes in large market centers in each sector. (a) Agricultural product sector. (b) Textiles and related sectors. (c) Cosmetics and beauty salons, electronics, home appliance, and other sectors. (d) Food service sector.

Given the variety of solid waste types created, the waste management strategy emphasizes the necessity for sector-specific techniques in recognition of the various business sectors found within significant market centers as shown in Table 10. The benefit is that it allows for a more efficient waste management procedure by concentrating similar firms nearby. It is possible to efficiently categorize solid waste kinds due to their homogeneity. The trade sectors have been carefully separated into distinct groups, which include the food service sector, the textile and allied enterprises, the agricultural product sector, and a combined category for other industries such as electronics, home appliances, and cosmetics and beauty salons, as shown in Figure 6. Because of the sector-specific segmentation, a customized approach to waste management is made possible, guaranteeing that the distinct qualities of every kind of organization are taken into account for efficient and long-lasting waste management in the major market centers in Bahir Dar City.

### 3.6. SWM Plan for Small Market Centers

Organic waste makes up the majority of solid waste in small market centers that are mostly agricultural products. These smaller centers' waste management plans are designed to address this main issue, placing a significant emphasis on the effective treatment and disposal of organic waste. Furthermore, a significant amount of plastic waste is produced, which calls for special consideration in the waste management strategy.

**Figure 7:**
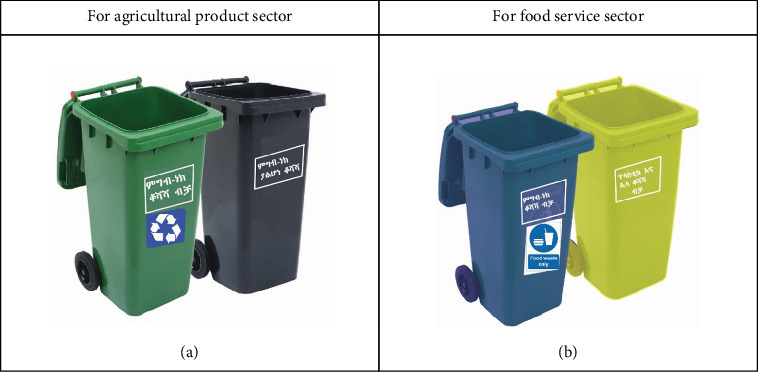
Labeled with local language (Amharic) trashcans for the collection of solid wastes in small market centers. (a) Agricultural product sector. (b) Food service sector.

Even though other wastes, such as hair and similar materials, are produced in lower quantities, the plan recognizes them and provides guidelines for handling them properly. The waste management plan for small market centers primarily addresses the efficient handling of organic and plastic waste, taking into account the unique waste-generating features of these markets for agricultural products as indicated in Table 11 and Figure 7.

## 4. Conclusions

In this study, a quantitative and qualitative analysis of the solid wastes generated in the main market centers of Bahir Dar City was carried out. The result indicated that a sector-specific waste management plan was very important since spatial differences are affected by economic activity, business types, and population density. There were significant differences in the current SWM methods between the market centers in Bahir Dar City; only two centers receive regular and separate collections. Moreover, the results indicated that food waste was the dominant waste type in Kebele 04 market centers, which stands out with almost half of the total waste generation (47%). On the other hand, retail trade emerges as the leading sector, generating nearly half of the total solid waste (49%, “Silassie” market center). The waste segregation indicated that wide range of possibilities to effectively reuse and recycle the solid waste for a value-added end use such as energy production, composting, and biogas production.. However, difficulties in the handling and collection stage, such as incomplete waste collecting containers and delays in waste removal, provide barriers to effective waste management. The main problems with SWM were addressed by the suggested waste management plan, which is based on a circular economy strategy.

SWM's theoretical contribution resides in its emphasis on creating plans to reduce the negative effects of waste generation on the environment and human health by encouraging resource conservation, cutting pollution, and improving waste disposal techniques. The ultimate goal is to achieve sustainable practices by implementing concepts like waste reduction, reuse, recycling, and recovery while taking into account the social and economic factors that affect waste generation and management practices.

As a guideline, a multimodal strategy that tackles several facets of Bahir Dar City's urban growth is necessary for the proposed waste management plan to be successful. The suggested plan must be easily incorporated into the current set of local laws and ordinances. One of the most important suggestions for bolstering the human infrastructure in support of waste management initiatives is capacity building. A strong structure for ongoing evaluation enables prompt modifications and enhancements in response to shifting waste production trends and market conditions.

Essentially, SWM means actively managing waste to prevent negative consequences for people and the environment. Its practical implications include the direct impact on public health, environmental quality, and aesthetics through proper collection, treatment, and disposal of discarded materials; lowering the risk of disease transmission from pests breeding in waste; reducing pollution from landfills; and improving the general quality of life in a community.

Overall, the study offers a thorough and focused waste management plan in addition to providing insightful information about the condition of SWM in Bahir Dar City's marketplaces. This plan, which takes into account the varied nature of enterprises and is based on a circular economy approach, lays the groundwork for more effective, long-lasting, and sector-specific waste management techniques in the city. Furthermore, the results provide insightful information to stakeholders, legislators, and urban planners striving for effective and sustainable municipal SWM techniques in Bahir Dar and other comparable urban environments in Ethiopia and the globe as a whole. Above all, the suggested strategy must be smoothly incorporated into the current municipal laws and policies. Building capacity is a crucial suggestion for bolstering the human infrastructure and assisting with waste management initiatives. Identifying and developing waste conversion technologies are very crucial for future research with well-designed data collection mechanism.

## Figures and Tables

**Table 1:** Population size and strata categorization.

**Name of market centers**	**Population size**	**Size**	**Strata**	**Sampling area**
Kebele 04	4810	Large	Stratum 1	Kebele 04
Kebele 12	2030	Large		
Abaymado	1125	Medium		
Silassie	265	Small	Stratum 2	Silassie
K/mihret	468	Small		
Abunehara	479	Small		
Gaja Mesk	259	Small		

*Note: Source:* Data from the market center committee, government offices, and interviews with stakeholders.

**Table 2:** Sample size allocation for each stratum.

**Stratum**	**Total population size**	**Weight given proportionally (%)**	**Allocated sample size**
Kebele 04 market center	4810	94	32
Silassie market center	265	6	3 + 1^a^
Total	2800	100	36

^a^Remark: 4 sample tokens to get representative data.

**Table 3:** Sample size allocation for subdivisions of each stratum for Kebele 04 market center.

**Subdivision**	**Weight given proportionally (%)**	**Kebele 04 market center**
Wholesale trade	7.5	3
Retail trade	84.4	25
Foodservice	3.1	2
Others	5	2
Total	100	32

**Table 4:** Sample size allocation for subdivisions of each stratum for Silassie market center.

**Subdivision**	**Weight given proportionally (%)**	**Silassie market center**
Wholesale trade	—	—
Retail trade	96	3
Foodservice	4	1
Others	—	—
Total	100	4

**Table 5:** Quantity of solid waste generated in each trade sector (kilograms per day).

**Waste category**	**Kebele 04 market center**				**Silassie market center**	
	**Retail trade (kg/day)**	**Food services (kg/day)**	**Wholesale trade (kg/day)**	**Others (kg/day)**	**Retail trade (kg/day)**	**Food services (kg/day)**
Food	2202.41	1938.28	745.55	0.00	451.10	39.85
Yard	10.49	3.64	0.00	6.54	0.00	0.00
Plastic (recyclable)	70.21	188.91	23.65	2.94	3.63	0.00
Plastic (nonrecyclable)	57.23	15.50	7.51	4.36	3.05	0.17
Paper and cardboard	277.20	10.80	32.11	140.47	0.00	0.00
Textiles	270.36	0.00	0.00	0.00	0.00	0.00
Rubber and leather	2.42	0.00	0.00	0.00	0.00	0.00
Electronics	16.56	0.00	0.00	0.00	0.00	0.00
Glass	28.99	2.81	0.00	0.00	0.00	0.00
Metals	36.28	0.00	0.00	0.00	0.00	0.00
Others	98.03	0.00	9.30	1.10	1.09	0.00
Total	3070.17	2159.94	818.12	155.41	458.87	40.02

**Table 6:** Solid waste generation rate for each market center.

**Waste category**	**Daily solid waste generated in market centers (kg/day)**							**Daily total (kg/day)**	**Yearly total (kg/year)**
	**Kebele 04**	**Silassie**	**Gaja mesk**	**Abunehara**	**Kidanemihret**	**Abaymado**	**Kebele 12**		
Food	4886.23	490.96	479.84	887.43	867.05	1142.83	2062.17	10,816.51	3,948,024.92
Yard	20.66	0.00	0.00	0.00	0.00	4.83	8.72	34.22	12,489.55
Plastic (recyclable)	285.71	3.63	3.55	6.56	6.41	66.82	120.58	493.25	180,037.22
Plastic (nonrecyclable)	84.60	3.22	3.14	5.81	5.68	19.79	35.70	157.95	57,650.25
Paper and cardboard	460.58	0.00	0.00	0.00	0.00	107.72	194.38	762.68	278,379.64
Textiles	270.36	0.00	0.00	0.00	0.00	63.23	114.10	447.69	163,408.04
Rubber and leather	2.42	0.00	0.00	0.00	0.00	0.57	1.02	4.00	1460.30
Electronics	16.56	0.00	0.00	0.00	0.00	3.87	6.99	27.43	10,010.39
Glass	31.80	0.00	0.00	0.00	0.00	7.44	13.42	52.66	19,221.88
Metals	36.28	0.00	0.00	0.00	0.00	8.48	15.31	60.07	21,926.47
Others	108.43	1.09	1.06	1.97	1.92	25.36	45.76	185.60	677,44.35
Daily total	6203.64	498.89	487.59	901.77	881.06	1450.95	2618.17	13,042.06	
Yearly total	2,264,327.46	18,2094.49	177,971.59	329,144.37	321,585.73	529,598.42	955,630.93	4,760,353.00	

**Table 7:** Solid waste composition (kilograms per day) of Silassie market center.

**Waste category**	**Retail trade**			**Average**
	**Agricultural products**	**Pepper, spices, and related**	**Food services**	
Food				
Food inputs	3.483	0.0692	2.759	2.104
Food leftovers	0		0.864	0.432
Yard	0	0	0	0
Plastics				
Recyclable	0	0.0286	0	0.010
Nonrecyclable	0.024	0	0.015	0.0131
Paper and cardboard	0	0	0	0
Textiles	0	0	0	0
Rubber and leather	0	0	0	0
Electronics	0	0	0	0
Glass	0	0	0	0
Metals	0	0	0	0
Others	0.0085	0	0	0.0029

**Table 8:** Solid waste composition (kilograms per day) data of Kebele 04 market center.

**Waste category**	**Waste source**													**Average**
	**Retail trades**							**Food services**		**Wholesale trade**			**Others**	
	**Agricultural products**	**Textiles and related**	**Cosmetics**	**Beauty salons**	**Electronics**	**Home appliances**	**Pepper, spices, and related**	**Large**	**Medium**	**Cosmetic and beauty salons**	**Textiles and related**	**Agricultural products**	**Game houses**	
Food														
Food inputs	4.32	0	0	0	0	0	0.021	5.3	5.769	0	0	0	0	1.185
Food leftovers	0	0	0	0	0	0	0	11.413	3.536	0	0	6.196	0	1.626
Yard		0.010	0	0	0	0.007	0	0	0.049	0	0	0	0	0.005
Plastics														
Recyclable	0	0.007	0	0	0.010	0.020	0.007	2.163	0.133	0	0.166	0.023	0	0.194
Nonrecyclable	0.003	0.005	0.021	0.025	0.003	0.008	0.003	0.240	0.024	0.008	0.025	0	0.001	0.028
Paper and cardboard	0	0.005	0.059	0.052	0.326	0.050	0.006	0.129	0.016	0.189	0.078	0	0.583	0.115
Textiles	0	0.533	0	0	0	0	0	0	0	0	0	0	0	0.041
Rubber and leather	0	0.005	0	0	0	0	0	0	0	0	0	0	0	0.000
Electronics	0	0	0	0	0.033	0	0	0	0	0	0	0	0	0.003
Glass	0	0	0	0	0.089	0.057	0	0.023	0.014	0	0	0	0	0.014
Metals	0	0	0	0	0.072	0	0	0	0	0	0	0	0	0.006
Others	0	0	0	0.189	0.004	0	0	0	0	0	0.041	0.036	0.005	0.021

**Table 9:** Average proximate analysis result of different solid waste in the market center.

**Waste**	**Moisture content (%)**	**Volatile matter (%)**	**Ash content (%)**	**Fixed carbon (%)**
Food waste	63.25	10.12	10.87	16.0.85
Plastic	10.53	68.95	4.24	16.94
Paper	13.94	46.51	5.68	34.12
Textile	17.14	39.11	7.47	33.13
Yard waste	40.14	23.43	8.87	26.82
Average	145	188.12	28.269	127.86

**Table 10:** Sector-specific solid waste management plan for larger market centers.

**Trade sectors**	**Handling and separation, storage, and processing at source mechanism**	**Collection mechanism**	**Transfer and transport**
Agricultural product	- Clearly labeled bins (Figure 6a) - Convenient placement—put the container in junctions to make it accessible - 2 containers needed at the same place: 1—for organic wastes (based on the daily waste composition generation food waste is dominant) 1—for other wastes - Container made from HDPE	○ Investment in collection infrastructure: allocate resources for the improvement and expansion of waste collection infrastructure, including the provision of adequate collection bins, vehicles, and equipment ○ Establish collection points: set up designated collection points within the market centers for different types of waste (e.g., recyclables, organics, and nonrecyclables) ○ Regular collection schedule: ◾ Implement a well-organized and frequent waste collection schedule to prevent overflow and ensure that sorted waste is promptly removed ◾ Collaborate with local waste collection services to optimize scheduling ○ Training programs: develop comprehensive training programs for informal waste collectors on effective waste sorting techniques, emphasizing the economic benefits of collecting valuable materials ○ Technology integration: if there is financial capacity, the use of technology, such as GPS tracking and route optimization software, streamlines and monitors waste collection activities ○ Public–private partnerships: foster collaborations between public authorities and private waste management entities to combine resources, expertise, and technology	○ Establish a formal collaboration with Dream Light PLC to integrate their existing waste collection for composting and charcoal briquette into the market's waste management system • Disposable waste transport to the disposal site
Textiles and related	- Clearly labeled bins (Figure 6b) - Convenient placement—put the container in junctions to make it accessible - 3 containers needed at the same place: 1—textile scraps (based on the daily waste composition generation textile product waste is dominant) 1—recyclable wastes 2—nonrecyclable wastes - Container made from HDPE		• For reusable wastes partner closing and textile product traders with individuals in need of their waste • Disposable waste transport to the disposal site • Separate the wastes in the recyclable containers sell give them to the currently active vendors in the area mostly cleaners and other individuals
Cosmetics and beauty salons, electronics, home appliances, others	- The nature of solid waste generated in these sectors is similar (plastic, cardboard, and glass in small amounts) - Clearly labeled bins (Figure 6c) - Convenient placement- put the container in junctions to make it accessible 2 containers in the same place: 1—recyclable wastes 1—nonrecyclable wastes		• Separate the wastes in the recyclable containers sell give them to the currently active vendors in the area mostly cleaners and other individuals • For nonrecyclable wastes haul them on waste transport vehicles on schedule
Food services	- Food leftovers and food scraps during preparation are the dominant types of solid waste. Plastic, paper, and cardboard are next, and little glass and yard waste -3 containers needed at the same place: 1—for food leftovers 1—for food waste during preparation 1—for plastic and other wastes - Clearly labeled bins (Figure 6d)		• Donate edible food scraps to food banks or composting facilities. • Establish a formal collaboration with Dream Light P.L.C. to integrate their existing waste collection for composting the food waste during preparation. • Separate the recyclable wastes and give them to the currently active vendors in the area mostly cleaners and other individuals.

**Table 11:** Solid waste management mechanism for small market centers.

**Trade sector**	**Handling and separation, storage, and processing at source mechanism**	**Collection mechanism**	**Transfer and transport**
Agricultural product sector	- Clearly labeled bins (Figure 7a) - Convenient placement- put the container in junctions to make it accessible - 2 containers needed at the same place: 1—for organic wastes (based on the daily waste composition generation food waste is dominant) 1—for other wastes - Container made from HDPE	The solid waste collection mechanism is the same as the larger market centers	• Establish a formal collaboration with Dream Light PLC to integrate their existing waste collection for composting and charcoal briquette into the market's waste management system • Disposable waste transport to the disposal site
Food services	- Clearly labeled bins (Figure 7b) - Food leftovers and food scraps during preparation are the dominant types of solid waste. Food leftovers in those market centers are not edible to eat. - 2 containers needed at the same place 1—for organic waste 1—for plastic and others		• Separate the recyclable wastes and give them to the currently active vendors in the area mostly cleaners and other individuals • Establish a formal collaboration with Dream Light PLC to integrate their existing waste collection for composting the food waste during preparation

## Data Availability

The data that support the findings of this study can be obtained from the corresponding authors on request.
